# Kongressbericht 11. Weltkongress Melanom und 21. Kongress der European Association of Dermatooncology in Athen

**DOI:** 10.1111/ddg.15898_g

**Published:** 2025-10-23

**Authors:** Ulrike Leiter, Ralf Gutzmer

Vom 2.4.–5.4. 2025 fand der 11. Weltkongress Melanom in Assoziation mit dem 21. Kongress der European Association of Dermatooncology (EADO) in Athen statt.

Der EADO Vorstand, deren Vorsitz seit 2015 Prof. Claus Garbe inne hatte und der diesen maßgebend geprägt hat, wurde neu gewählt. Der neue Vorsitzende ist nun Prof. Josep Malvehy aus Barcelona.

Tagungspräsident Prof. Alexander Stratigos (Athen) sowie die Kongressleitung (Prof. Claus Garbe (Tübingen) und Prof. Axel Hauschild (Kiel)), der zudem als Präsident der World Melanoma Society vorsteht, betonten den wichtigen wissenschaftlichen Austausch in der Dermatoonkologie weltweit. Der EADO‐Kongress ist der größte europäische Hauttumor‐ Kongress, an dem in diesem Jahr 1997 Teilnehmer aus 60 Ländern registriert waren. Die 127 Mitglieder der Fakultät stellten ein hochkarätiges Programm zusammen, das 7 herausragende Keynote Lectures beinhaltete. Namhafte Redner präsentierten in 79 Sitzungen, 65 Symposien und 8 Industrie gesponserten Satellitensymposien eine breite Palette dermatoonkologischer Themen. Zudem wurden 443 Abstracts eingereicht, die den Kongress mit 62 Freien Vorträgen und 362 Posterpräsentationen bereicherten.

Mit einem überaus vielfältigen Kongressprogramm wurden neueste wissenschaftliche Erkenntnisse und aktuelle Studien mit einem dermatoonkologischen Update in Epidemiologie, Prävention, Diagnostik und Therapie vorgestellt. Hierbei wurden aktuelle Therapien und Studientherapien im fortgeschrittenen Stadium, in der Adjuvanz und im neoadjuvanten Konzept diskutiert, Ausblicke auf künftige Behandlungen vorgestellt, das Management von Nebenwirkungen sowie die Nachsorge verschiedener Tumorentitäten adressiert.

Diskutiert wurden die Epidemiologie des Melanoms und nichtmelanozytärer Hauttumore in Europa, Nordamerika und Australien, hier zeigt sich, dass vor allem bei älteren Patienten steigende Inzidenzraten und nach wie vor hohe Mortalitätsraten zu erwarten sind. Bei kutanen Plattenepithelkazinomen zeigen sich ungebrochen steigende Inzidenzraten, vor allem in West‐ und Nordeuropa, den USA und Australien, die zudem in Australien die dritthöchsten Kosten aller Tumore verursachen. Auch hier finden sich die höchsten Inzidenzraten bei älteren Patienten mit zunehmender Belastung für die Gesundheitssysteme weltweit. In diesem Zusammenhang wurden Diagnostik, Screening (auch unter dem Einsatz künstlicher Intelligenz), prognostische Bio‐ Marker und die Nachsorge des Melanoms in kontroversen Sitzungen kritisch diskutiert. Hier wurde ein großer Bedarf festgestellt, Strategien zu etablieren, die Gruppen mit dem höchsten Risiko zu identifizieren und diese einer engmaschigen Beobachtung zuzuführen.

Ein weiterer Schwerpunkt stellte die neoadjuvante Therapie dar. Hier ist ein Paradigmenwechsel für das metastasierte Melanom im Stadium III zu erwarten. Mehrere Studien wie die Prado Studie und die Opacin Neo Studie hatten vorab einen Nutzen der neoadjuvanten Therapie gezeigt. Im letzten Jahr wurde in der Nadina Studie nach zwei Zyklen Ipilimumab Nivolumab im neoadjuvanten Setting bei Patienten im Stadium III (makroskopische Metastasen) ein deutlicher Vorteil im rezidivfreien Überleben gezeigt. Nach zwei Zyklen zeigten Patienten in der neoadjuvant behandelten Gruppe in 59.0% eine major pathological response (MPR, < 10% vitale Tumorzellen) Bei diesen Patienten betrug das rezidivfreie Überleben nach zwölf Monaten 95%, auf eine weitere adjuvante Therapie kann dann verzichtet werden. In den Niederlanden wird diese Therapie zwischenzeitlich vergütet.

Eine neoadjuvante Studie, die eine intraläsionale Immuntherapie bei Patienten mit makroskopischen Lymphknoten‐ oder Intransitmetasasen vorsieht, zeigte ebenfalls positive Daten. In der Pivotal Studie erhielten Patienten vier intraläsionale Injektionen mit Daromun, einem Gemisch aus an einen Fibronektin‐Antikörper gebundenes Interleukin‐2 und TNF‐alpha. Im Kontrollarm erhielten die Patienten den Therapiestandard, das heißt eine Resektion beziehungsweise Lymphknotendissektion. In beiden Armen konnte eine adjuvante Therapie wie zugelassen angeschlossen werden. In der Effektivität zeigte sich für den neoadjuvanten Arm im RFS ein signifikanter Vorteil gegenüber Standardtherapie mit einem absoluten Unterschied von 15% nach drei Jahren (HR 0,59; 95%CI 0,41‐0,86)

Eine weitere Option für die Erstlinie des fortgeschrittenen Melanoms stellt der adoptive T‐Zell Transfer (TIL Therapie) dar. In den USA wurde diese Therapie in der Erstlinie bereits zugelassen, in Europa wurde die Zulassung beantragt. In Deutschland sind erste Zentren geplant, beispielsweise am Universitätsklinikum Hamburg. Als zusätzliche Therapieoption erhielt Replimune den Status einer bahnbrechenden Therapie für RP1 in Kombination mit Nivolumab zur Behandlung von fortgeschrittenem Melanom, basierend auf der Sicherheit und klinischen Aktivität, die in der Melanom‐Kohorte des IGNYTE‐Programms beobachtet wurde, die nicht auf Anti‐PD‐1 angesprochen hatte. Eine Bestätigung Phase 3‐Studie IGNYTE‐3 ist derzeit im Gange und weltweit sind über 100 Standorte geplant.

Dies konnte nur eine Auswahl interessanter Beiträge vom EADO/World Melanom Kongress sein. Es gab viele weitere interessante Studien, Real‐World Daten und Educationals.

Die EADO trat erneut als eine starke Europäische Gesellschaft in Erscheinung und der Besuch des Kongresses war aus dermatoonkologischer Sicht eine auf jeden Fall eine Reise wert!

## DANKSAGUNG

Open access Veröffentlichung ermöglicht und organisiert durch Projekt DEAL.

